# Histone deacetylase inhibitor induces cell apoptosis and cycle arrest in lung cancer cells via mitochondrial injury and p53 up-acetylation

**DOI:** 10.1007/s10565-016-9347-8

**Published:** 2016-07-16

**Authors:** Lianmin Bao, Hua Diao, Nian Dong, Xiaoqiong Su, Bingbin Wang, Qiongya Mo, Heguo Yu, Xiangdong Wang, Chengshui Chen

**Affiliations:** 1 Department of Respiratory and Critical Care Medicine, The First Affiliated Hospital of Wenzhou Medical University, Wenzhou, 325000 Zhejiang China; 2 Key Laboratory of Reproduction Regulation of NPFPC, SIPPR, IRD, Fudan University, Shanghai, 200032 China

**Keywords:** Acetylation, Apoptosis, Epithelial-mesenchymal transition, Mitochondria, p53, Histone deacetylase inhibitor

## Abstract

The reversibility of non-genotoxic phenotypic changes has been explored in order to develop novel preventive and therapeutic approaches for cancer. Quisinostat (JNJ-26481585), a novel second-generation histone deacetylase inhibitor (HDACi), has efficient therapeutic actions on non-small cell lung cancer (NSCLC) cell. The present study aims at investigating underlying molecular mechanisms involved in the therapeutic activity of quisinostat on NSCLC cells. We found that quisinostat significantly inhibited A549 cell proliferation in dose- and time-dependent manners. Up-acetylation of histones H3 and H4 and non-histone protein α-tubulin was induced by quisinostat treatment in a nanomolar concentration. We also demonstrated that quisinostat increased reactive oxygen species (ROS) production and destroyed mitochondrial membrane potential (ΔΨm), inducing mitochondria-mediated cell apoptosis. Furthermore, exposure of A549 cells to quisinostat significantly suppressed cell migration by inhibiting epithelial-mesenchymal transition (EMT) process. Bioinformatics analysis indicated that effects of quisinostat on NSCLC cells were associated with activated p53 signaling pathway. We found that quisinostat increased p53 acetylation at K382/K373 sites, upregulated the expression of p21^(Waf1/Cip1)^, and resulted in G1 phase arrest. Thus, our results suggest that the histone deacetylase can be a therapeutic target of NSCLC to discover and develop a new category of therapy for lung cancer.

**Electronic supplementary material:**

The online version of this article (doi:10.1007/s10565-016-9347-8) contains supplementary material, which is available to authorized users.

## Introduction

Lung cancer is the leading cause of cancer-related mortality worldwide with an incidence of about 1.5 million people each year and an overall 5-year survival rate less than 16 % (Tartour and Zitvogel 2013). According to histological classifications, non-small cell lung cancer (NSCLC) represents approximately 80 % of all lung cancers. In the last decade, the major advance in the treatment of NSCLC grew from the recognition that specific genetic alterations define subsets of NSCLC (Berge and Doebele 2014), such as epidermal growth factor receptor (EGFR) mutations, Kirsten rat sarcoma viral oncogene homolog (KRAS) mutations, and anaplastic lymphoma kinase (ALK) translocation, with a number of drugs targeting such specific oncogenes. Despite these advancements, loss of treatment efficacy with acquired resistance is becoming universal in NSCLC patients treated with these targeted agents (Camidge et al. 2014). New targets are therefore needed to improve the efficacy of lung cancer therapy.

In addition to genetic alterations, epigenetic changes may also contribute to the development and progression of solid and hematologic malignancies (Lund 2004). Generally speaking, acetylation modification of “histone tails” is the most extensively studied epigenetic event, which regulates transcriptional activities and affects biological processes and functions (Allfrey et al. 1964). Histone acetylation and deacetylation are controlled by two classes of enzymes—histone acetyltransferases (HATs) and histone deacetylases (HDACs) (Kurdistani and Grunstein 2003). HDACs are known to be intimately related with the development and progression of cancers (Weichert 2009), including lung cancer. It is reported that HDACs are frequently overexpressed in lung cancer, and this overexpression correlates with poor prognosis and drug resistance (Osada et al. 2004). Thus, in contrast to genetic cancer causes, HDACs and epigenetic changes may provide new targets for therapeutic intervention in NSCLC.

Histone deacetylase inhibitors (HDACis) have emerged as a novel class of anti-cancer agents that regulate chromatin structure and gene expression, inducing growth inhibition, apoptosis, and differentiation in various tumor cells (Kelly and Marks 2005; Liu et al. 2006; Miller et al. 2011; Zhang and Zhong 2014). HDACis significantly increase core histone acetylation, resulting in increased expression of several genes that are often silenced in cancer, such as tumor suppressor genes (Liu et al. 2006). Despite histones, a wide variety of non-histone proteins are also demonstrated to be regulated by acetylation, which may contribute to the anti-tumor effects of HDACi (Zhang and Zhong 2014). In this study, we investigated the potential therapeutic value of quisinostat (JNJ-26481585), a novel second-generation HDACi, in NSCLC cells. In vitro, quisinostat has shown anti-proliferative activity against NSCLC cell lines (Arts et al. 2009), while the molecular mechanisms are still unclear. Our results showed that quisinostat extremely changed protein acetylation patterns of A549 cells and inhibited epithelial-mesenchymal transition (EMT) process. Exposure to quisinostat induced mitochondrial injury and increased p53 acetylation, resulting in cell apoptosis and G1 phase arrest. In conclusion, these findings provide the proof of concept for evaluation of quisinostat as a novel NSCLC chemotherapeutic agent.

## Materials and methods

### Cell culture

Human NSCLC cell line A549 was obtained from the Cell Bank of the Chinese Academy of Sciences (Shanghai, China). Cells were cultured in high-glucose DMEM (Gibco, Invitrogen, UK) containing 10 % fetal bovine serum (FBS) (Gibco, Invitrogen, UK) and 100 U/ml of penicillin G/streptomycin. These cells were cultured in an incubator at 37 °C in 5 % CO_2_ with a humidified atmosphere.

### Cell viability assay

**Fig. 1:**
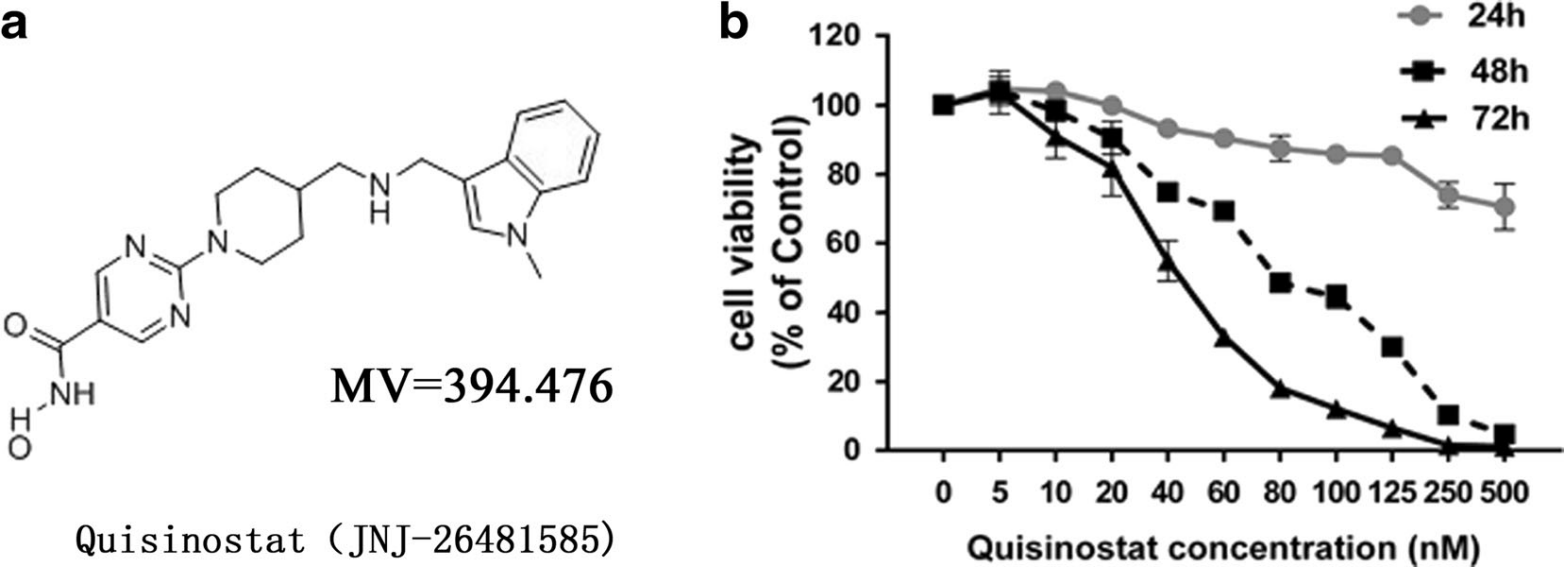
Effect of quisinostat on the viability of A549 cells. **a** Chemical structure of quisinostat (JNJ-26481585). **b** A549 cells were treated with series of quisinostat for 24, 48, or 72 h, and cell viability was evaluated. Mean ± SD of three experiments performed in triplicate is shown

Cell Counting Kit-8 (CCK-8) (Beyotime, China) was utilized to evaluate cell viability of cells treated with quisinostat (the chemical structure of quisinostat is shown in Fig. 1a). The cell samples were exposed to various concentrations of quisinostat for 24, 48, or 72 h. After the end of incubation, the medium was removed from each well, 10 μl of CCK-8 and 100 μl of serum-free medium were added, and the cells then were incubated for 1 h at 37 °C. The absorbance of each well was measured at 450 nm using a microplate reader (Thermo Fisher Scientific, USA). Each well was triplicate.

### Wound healing experiment

Wound healing experiment was performed as described previously (Lee et al. 2010). Photographs of the wound area were captured at 0, 24, and 48 h with a microscope (Nikon ECLIPSE TS100, Japan). Data were quantified through analyzing the areas in the scratch not covered by cells with ImageJ software version 1.49. Closure rate was measured as percentage of the area at 0 h.

### Cell migration experiment

Transwell chambers (Corning, NY, USA) were used in cell migration experiments as described previously (Chaulet et al. 2001). Cells were incubated at 37 °C for 24 h, and cells that did not penetrate the polycarbonate membrane at the bottom of the upper chamber were erased with cotton swabs. The cells that penetrated through the membrane were fixed with methanol and 0.1 % crystal violet for 10 min at room temperature. The stained crystal violet was eluted with 33 % acetic acid solution. The optical density (OD) value of the solution was detected with a microplate reader at 570 nm. The migration activity was quantified by cell counter associated with the OD value.

### Intracellular ROS measurement

Intracellular reactive oxygen species (ROS) was measured by use of a 2′,7′-dichlorodihydrofluorescein diacetate (DCFH-DA) probe (Sigma, USA). Pretreated with various concentrations of quisinostat for 24 h, A549 cells were exposed to serum-free medium containing 10 μM DCFH-DA for 30 min in the darkness. Then, the cells were washed with the medium for three times, and fluorescent intensity was measured by flow cytometry. Data were analyzed using FlowJo software vesion 7.6.

### Assessment of mitochondrial membrane potential (ΔΨm)

Cells were stained with ΔΨm-sensitive probe JC-1 (5,5′,6,6′-tetrachloro-1,1′,3,3′-tetraethyl-imidacarbocyanine iodide) (Beyotime, China), at a final concentration of 5 μg/ml in serum-free medium for 20 min at 37 °C in the darkness. After PBS washes, JC-1-labeled cells were observed by a confocal microscope (Olympus, USA) and harvested cells were analyzed by flow cytometry. Increased green fluorescence and deceased red fluorescence of the cells were observed as the mitochondrial membrane potential collapsed.

### Intracellular ATP measurement

To measure intracellular ATP levels, an ATP detection kit (Beyotime, China) was used. Pretreated with quisinostat for 24 h, A549 cells were analyzed following the manufacturer’s instructions. The relative ATP level was calculated according to the following formula: relative ATP level (nmol/mg) = ATP value (nmol/ml) / protein value (mg/ml).

### Apoptosis analysis

Quisinostat-pretreated cells were trypsinized and washed with complete medium. The samples (5 × 10^5^ cells) were centrifuged for 5 min at 400×*g*, and the supernatant was discarded. The cells were then stained using an annexin V-fluorescein isothiocyanate (FITC)/propidium iodide (PI) solution from apoptosis kit (Beyotime, China) for 10 min in the darkness. The number of apoptotic cells was detected and analyzed using flow cytometry.

### Gene expression profiling and bioinformatics analysis

Microarray experiments were carried out using the Illumina BeadChip according to the manufacturer’s protocol. The raw data were analyzed using GenomeStudio and normalized according to the quantile algorithm. A two-tail *t* test, assuming unequal variance between the groups, was performed in order to determine significance. *p* value of 0.05 and diffscore of 20 were used to identify genes that were differentially expressed. Gene ontology (GO) (Ashburner et al. 2000) enrichment analysis was performed on the significant genes using the Database for Annotation, Visualization, and Integrated Discovery (DAVID) bioinformatics online toolset (da Huang et al. 2009). Additionally, enrichment was also performed on pathways from the Kyoto Encyclopedia of Genes and Genomes (KEGG) (Kanehisa et al. 2004).

### Cell cycle analysis

We performed cell cycle analysis using PI (Sigma-Aldrich) staining, followed by flow cytometry as previously described (Zhu et al. 2015). Data were evaluated using ModFit LT version 3.1.

### Real-time reverse transcription polymerase chain reaction

Total RNA of A549 cells was extracted using TRIzol (Invitrogen, UK) following the protocol. Complementary DNA (cDNA) was synthesized in accordance with the manufacturer’s instructions (Toyobo, Japan). Quantitative normalization of cDNA in each sample was performed using housekeeping gene glyceraldehyde-3-phosphate dehydrogenase (GAPDH) as an internal control to determine the uniformity of the template RNA for all specimens.

### Western blot assay

After 24 h of treatment with quisinostat, the cells were subjected to protein extraction. Sodium dodecyl sulfate-polyacrylamide gel electrophoresis and immunoblotting were performed as previously described (Yu et al. 2015).

### Statistical analysis

All data in this study were obtained from three independent experiments and then expressed as the means ± standard deviation (SD). Student’s *t* test was used to determine the difference between two groups. All the analysis was performed on SPSS 17.0 software (SPSS, IL, USA). The level of statistical significance was set at *p* < 0.05.

## Results

### Quisinostat inhibited the viability of A549 cells

The CCK-8 assay was used to evaluate the impact of quisinostat on cell viability. A549 cells were treated for 24, 48, or 72 h with quisinostat diluted to concentrations of 5, 10, 20, 40, 60, 80, 100, 125, 250, and 500 nM in complete medium. The results indicated that relative to the control cells, A549 cell exposure to quisinostat for 24 h exhibited viability of 104.6, 104.0, 99.8, 93.3, 90.5, 87.5, 85.8, 85.3, 74.0, and 70.5 %, respectively; cells treated for 48 h exhibited viability of 104.1, 98.4, 90.5, 74.8, 69.3, 48.6, 44.6, 30.0, 10.2, and 4.8 %, respectively; and cells treated for 72 h exhibited viability of 103.6, 91.0, 81.9, 54.8, 33.0, 18.1, 12.1, 6.5, 1.6, and 1.2 %, respectively (Fig. 1b). In addition, the IC_50_ values of cells for 48 and 72 h of quisinostat treatment were 82.4 and 42.0 nM, respectively. The assay results suggested that quisinostat extremely inhibited the proliferation of A549 cells in dose- and time-dependent manners (Fig. 1b). Meanwhile, we found that the viability of A549 cells did not change significantly with the dosage below 100 nM at 24-h time point. Therefore, the exposure concentration of quisinostat lower than 100 nM with exposure time at 24 h was chosen for further experiments.

### Quisinostat changed A549 protein acetylation patterns and increased acetylation of histones and α-tubulin

**Fig. 2:**
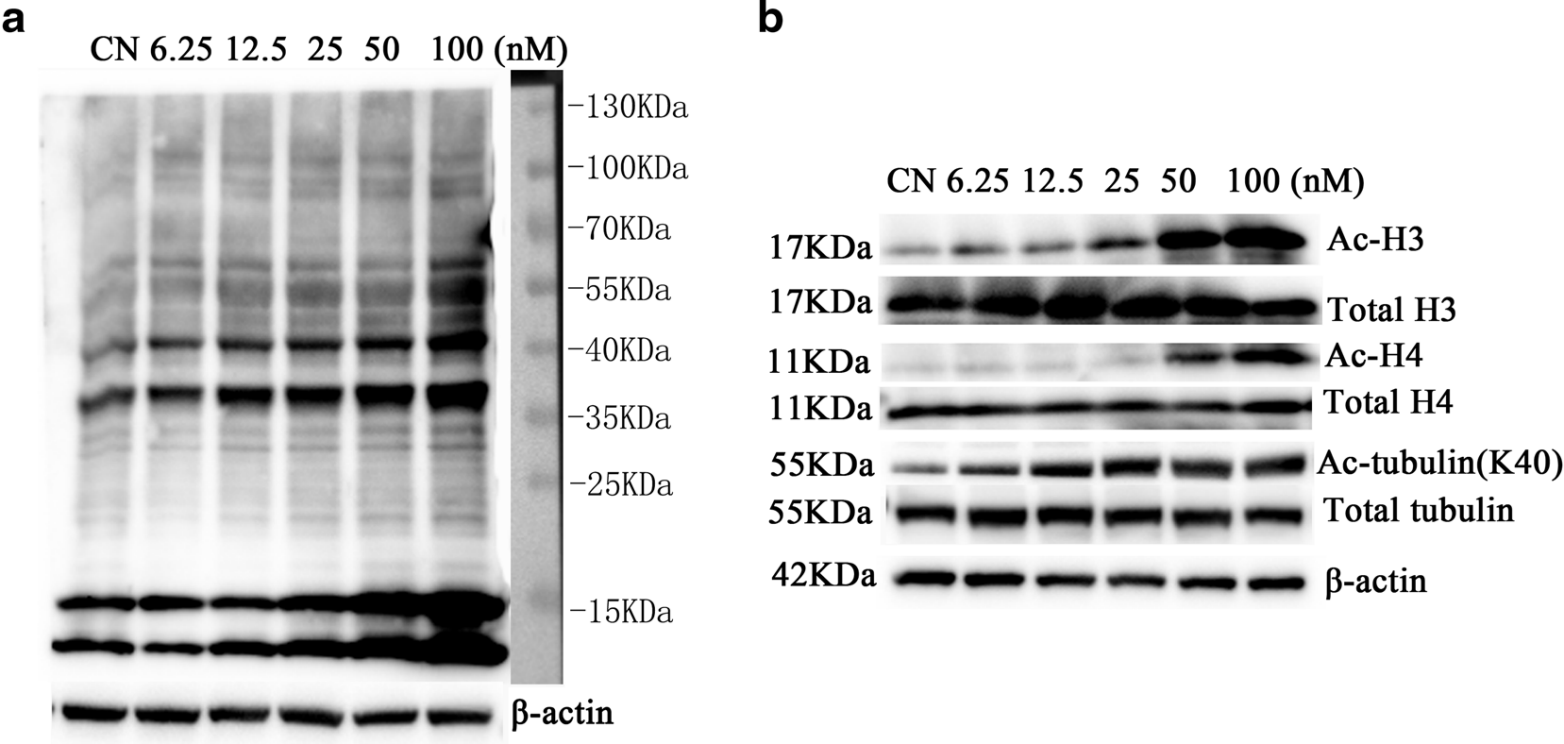
Effect of quisinostat on protein acetylation patterns. **a** A549 cells were treated with quisinostat for 24 h. Protein acetylation patterns were assessed by western blot (4–20 % gradient gel electrophoresis) using pan-anti-acetyllysine antibody. **b** A549 cells were treated with quisinostat for 24 h. Protein expression of acetylated histone 3 (Ac-H3), total H3, acetylated histone 4 (Ac-H4), total H4, acetylated α-tubulin (K40), α-tubulin, and β-actin was assessed by western blot

To identify acetylated proteins, we analyzed the cell lysates by western blot using gradient gel electrophoresis (4–20 %). After electrophoresis, the proteins were transferred to PVDF membranes and then immunoblotted with a pan-anti-acetyllysine monoclonal antibody (Yu et al. 2015). As shown in Fig. 2a, the antibody could detect the accumulation of acetylated proteins induced in A549 cells by treating with quisinostat. The differences in the density of the bands are thought to reflect the differences in the levels of protein acetylation. Although acetylated protein bands could not be defined accurately, we found extreme variation of acetylated patterns from quisinostat exposure to control (Fig. 2a). The most salient acetylated bands around 15-kDa position might be histones, which is consistent with the reports describing their acetylations (Graff and Tsai 2013). Another detected bands appeared at about 55 kDa; these acetylated proteins might be α-tubulin (Song and Brady 2015). The other bands should be further studied.

To validate our guess that quisinostat increased acetylation of histones and α-tubulin in A549 cells, we further detected these proteins using specific anti-acetylation antibodies. We found acetylation status of histones H3 and H4, and α-tubulin (K40) was significantly increased in a dose-dependent manner (Fig. 2b), which was consistent with our guess.

### Quisinostat inhibited A549 cell migration by suppressing EMT process

**Fig. 3:**
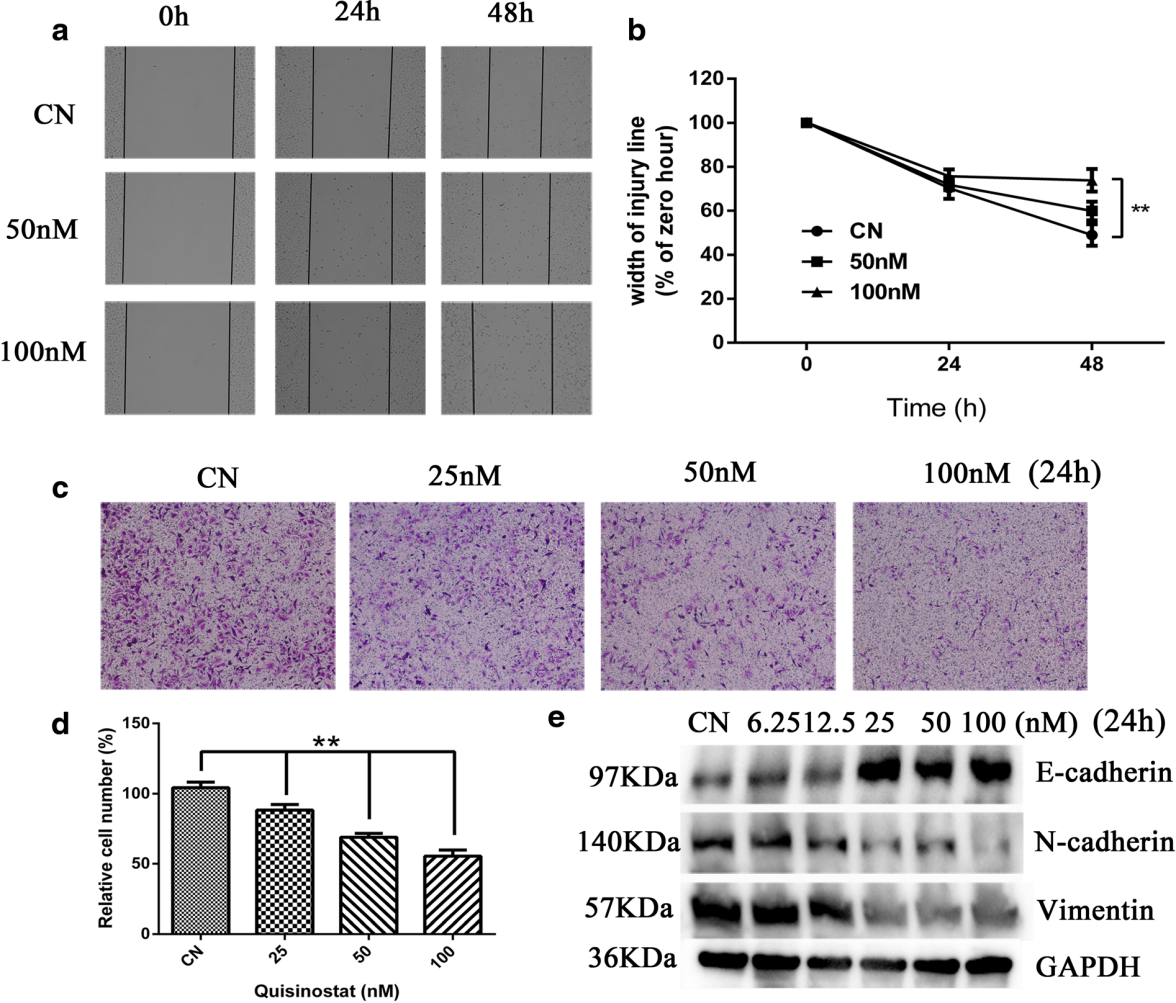
Effects of quisinostat on A549 cell migration and EMT process. **a** A549 cells were incubated in serum-free medium for 48 h, and cell migration was evaluated by wound healing assay (×40). **b** The wound width of five random views was measured, and the healing width was calculated. **c** Effect of quisinostat on A549 cell migration in a ×100 light scope after crystal violet staining by Transwell assay. **d** The absorbance of eluted crystal violet was read at 570 nm on a microplate reader. **e** A549 cells were treated with quisinostat for 24 h. Protein expression of E-cadherin, N-cadherin, Vimentin, and GAPDH was assessed by western blot. The results were obtained from triplicate experiments and represented as means ± SD (*n* = 3). *p < 0.05 and **p < 0.01 versus control group

Wound healing experiment was performed to evaluate the effect of quisinostat on A549 cell migration. As shown in Fig. 3a, b, more than half of the wound area in control group was recovered with cell monolayer after 48-h incubation compared with 0 h. The closure rates of groups treated with quisinostat at 50 and 100 nM were 59.92 ± 4.28 and 73.88 ± 5.12 %, respectively, which were significantly inhibited to the control group with 48.96 ± 4.88 % (*p* < 0.01) (Fig. 3b). Furthermore, in Transwell migration experiment, the number of migrating cells treated with quisinostat was significantly lower than that of control cells after 24 h of being cultured (Fig. 3c). The OD values of crystal violet suggested that migrating cells treated with quisinostat at 25, 50, and 100 nM were 88.30 ± 4.02, 68.97 ± 2.68, and 55.50 ± 4.33 % of control group cells (*p* < 0.01) (Fig. 3d).

To define the mechanism of anti-migration effect of quisinostat against A549 cells, we investigated the involvement of several EMT process signs. EMT process has been recognized as a key contributor in cancer cell migration and metastasis (Guarino et al. 2007), which is characterized by loss of cell adhesion, downregulation of E-cadherin expression, and acquisition of mesenchymal markers including N-cadherin and Vimentin to increase cell motility (Guarino et al. 2007). The western blot analysis data demonstrated that cells treated with quisinostat for 24 h upregulated E-cadherin expression (Fig. 3e) and downregulated N-cadherin and Vimentin expressions (Fig. 3e), indicating that quisinostat significantly inhibited A549 cell EMT process.

### Effects of quisinostat on the mitochondria

**Fig. 4:**
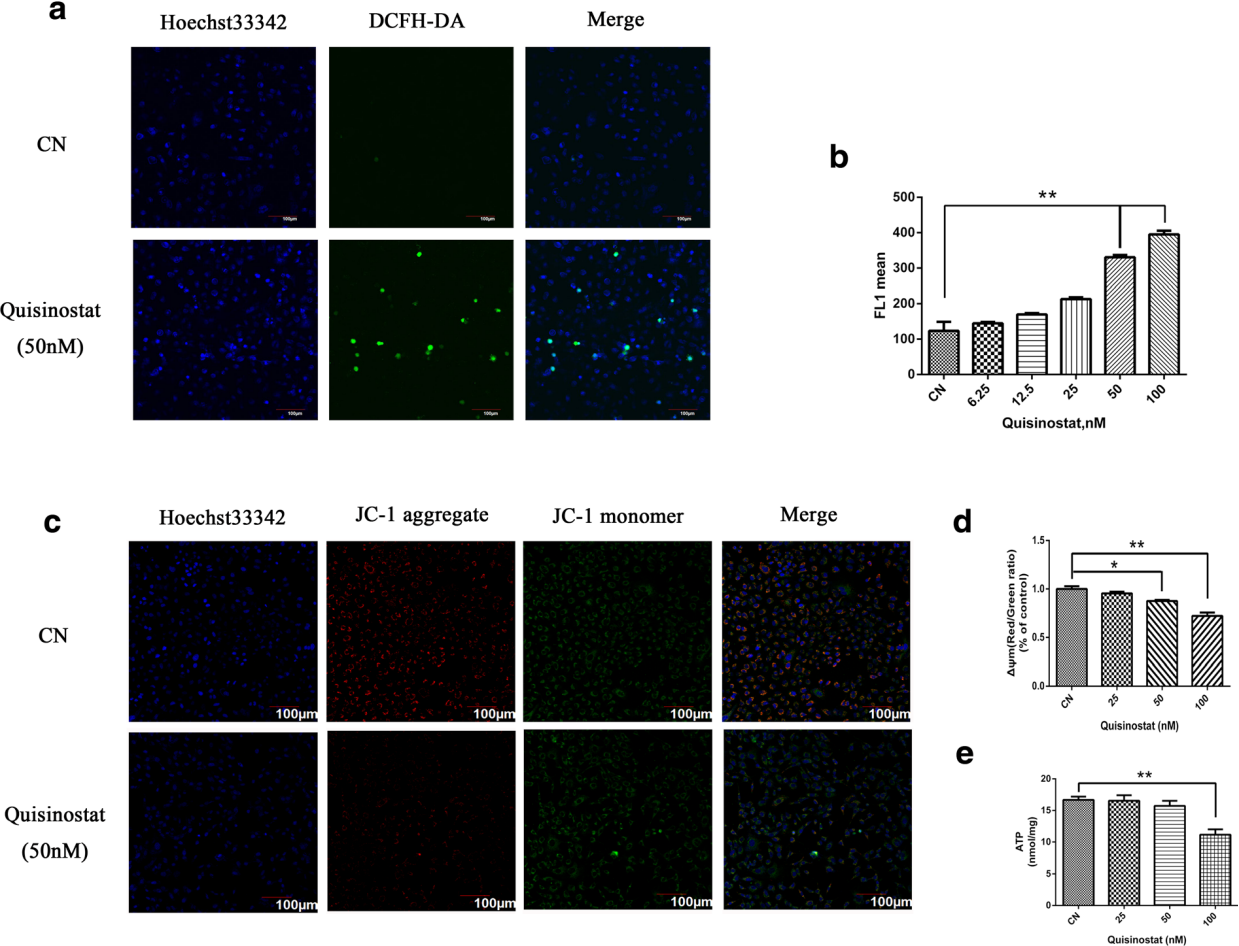
Effects of quisinostat on mitochondrial functions. **a** Representative images of A549 cells treated with quisinostat for 24 h versus normal and subjected to confocal microscopy of the DCFH-DA dye staining, as well as counterstaining with Hoechst 33342 (which marks chromatin). The increased intensity of cell fluorescence caused by treatment with quisinostat indicates elevated ROS production. **b** Flow cytometry analysis of intracellular ROS levels in A549 cells labeled with DCFH-DA dye. **c** A549 cells were treated with quisinostat for 24 h prior to JC-1 staining. Images shown are representative observations from three independent experiments. *Red images* indicate the JC-1 aggregate fluorescence from healthy mitochondria, while *green images* exhibit cytosolic JC-1 monomers. *Merged images* indicated the co-localization of JC-1 aggregates and monomers. **d** Mitochondrial potential loss assay by flow cytometry. **e** Effect of quisinostat on cellular ATP levels. Data are shown as mean ± SD, *n* = 3. *p < 0.05, **p < 0.01 versus control group (Color figure online)

Mitochondria are important in cell functions, and increasing evidences indicate that mitochondrial dysfunction is often involved in the induction of apoptosis (van Loo et al. 2002). To assess the role of mitochondria in anti-tumor effect of quisinostat, we tested whether mitochondrial ΔΨm will be collapsed by quisinostat treatment, which is an early marker of mitochondria-mediated apoptosis. Confocal microscopy observations showed that the red fluorescence was decreased and green fluorescence was increased with quisinostat treatment (Fig. 4c), indicating that mitochondrial ΔΨm was collapsed. Flow cytometry assay data also suggested that quisinostat caused an obvious decrease of ΔΨm in A549 cells. The red/green ratios of groups treated with quisinostat at 25, 50, and 100 nM were 95.46 ± 1.68, 87.60 ± 1.14, and 72.24 ± 3.40 %, respectively, compared with the control group (Fig. 4d).

Mitochondrial ΔΨm loss is often accompanied by the production of ROS (Vaux and Korsmeyer 1999). We found that quisinostat significantly induced ROS release in a dose-dependent manner (Fig. 4a, b). Cellular ATP depletion is another marker of early apoptosis; our results showed that high concentration of quisinostat significantly decreased intracellular ATP levels of A549 cells (Fig. 4e). These data indicated that quisinostat might trigger mitochondria-mediated apoptosis by increasing ROS production and decreasing ATP generation in A549 cells.

### Quisinostat induced mitochondria-mediated apoptosis

**Fig. 5:**
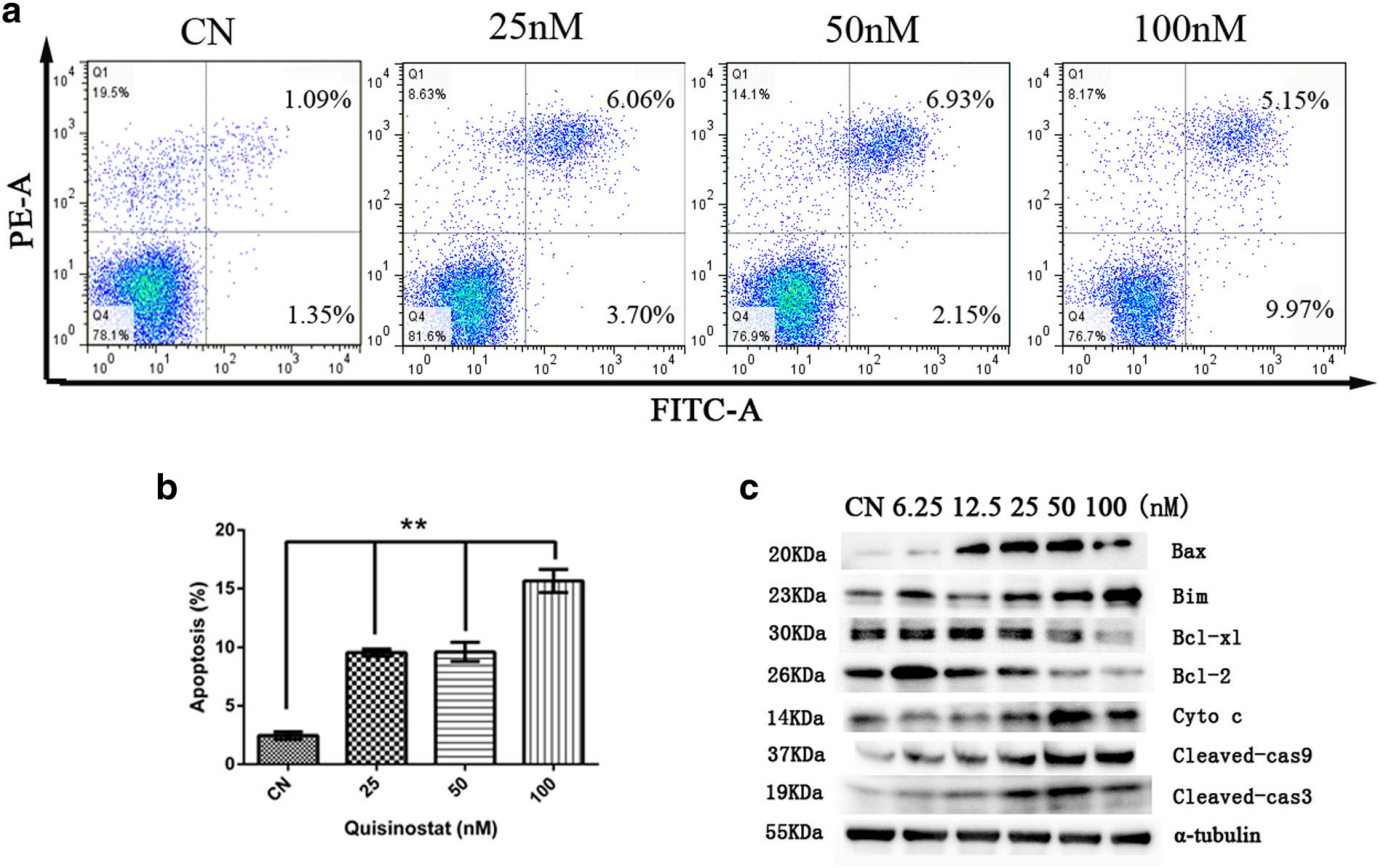
Apoptosis of A549 cells in vitro. **a** The cell apoptosis was determined by flow cytometry. The cells were treated with 25, 50, and 100 nm of quisinostat for 24 h, and then, cells were trypsinized, washed with PBS, and stained using an annexin V/FITC kit. Fluorescence intensity for annexin V/FITC is plotted on the *x*-axis, and PI is plotted on the *y*-axis. **b** Total annexin V/FITC-positive cells were analyzed as death cells. Data are shown as mean ± SD, *n* = 3. **p < 0.01 versus control group. **c** Protein expression of Bax, Bim, Bcl-xl, Bcl-2, Cyto c, cleaved caspase-9 (Cleaved-cas9), cleaved caspase-3 (Cleaved-cas3), and α-tubulin was assessed by western blot

To characterize quisinostat-induced apoptosis, A549 cells were stained with annexin V-FITC and PI and then analyzed by flow cytometry. The result showed that quisinostat increased apoptosis in A549 cells (Fig. 5a), and the percentages of apoptotic cells of control and 25, 50, and 100 nM were 2.65 ± 0.19, 9.75 ± 0.06, 9.28 ± 0.25, and 15.00 ± 0.17 %, respectively (Fig. 5b).

To define the molecular mechanisms, we investigated the involvement of Bcl-2 family proteins and caspase family proteins during the induction of cell apoptosis by quisinostat in A549 cells. We found that exposure to quisinostat resulted in a marked decrease in protein expressions of anti-apoptotic proteins Bcl-2 and Bcl-xl (Fig. 5c) and a drastic increase of pro-apoptotic proteins Bax and Bim (Fig. 5c) as well as cleaved caspase-9 and cleaved caspase-3 proteins (Fig. 5c). Moreover, we also found that Cyto c was significantly upregulated (Fig. 5c), which is involved in mitochondria-mediated apoptosis.

### Bioinformatics analysis indicated that quisinostat was involved in p53 signaling pathway activation

**Fig. 6:**
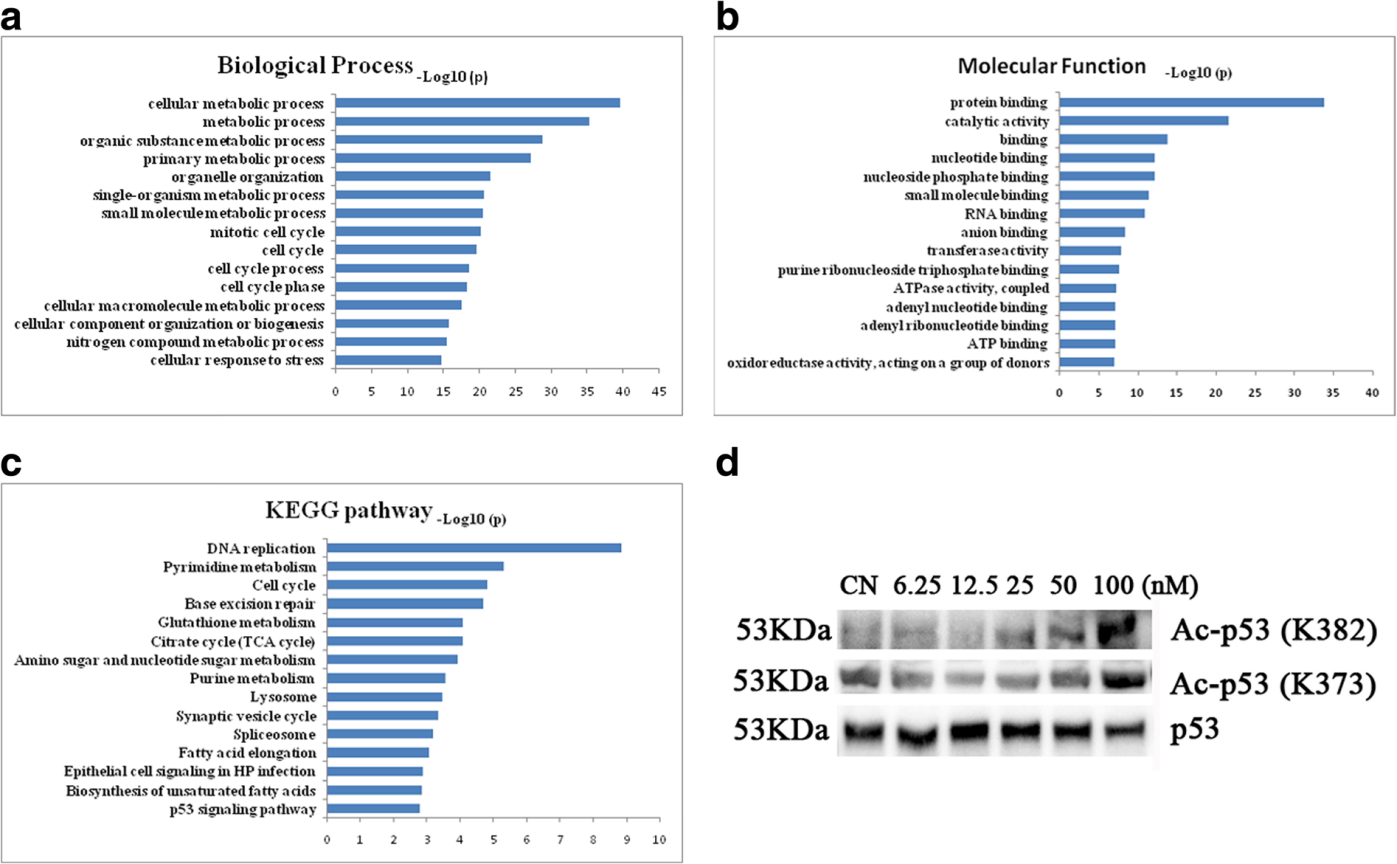
GO and KEGG analysis of gene expression profiling. **a** Biological process analysis. **b** Molecular function analysis. **c** KEGG pathway analysis. **d** A549 cells were treated with quisinostat for 24 h. Protein expression of acetylated p53 (Ac-p53) and p53 was assessed by western blot

To search for the pathways affected by quisinostat treatment, gene expression profiling was performed. After data collecting and normalizing, we compared the genes of quisinostat treatment group to controls followed by diffscore. Selected genes were further analyzed by bioinformatics methods including GO analysis and KEGG pathway analysis. GO analysis demonstrated that targeted genes were significantly enriched in cellular metabolic process, mitotic cell cycle, and cell cycle process in biological process (Fig. 6a); the terms of molecular function include protein binding, RNA binding, and ATP binding (Fig. 6b). In KEGG pathway analysis, similar terms were enriched, including DNA replication and cell cycle (Fig. 6c). Interestingly, we found that p53 signaling pathway was significantly enriched (Fig. 6c). According to the profiling results, the expression level of p53 was little changed, but many downstream genes of p53 were significantly altered (Supplement Fig. 1), indicating that p53 activity might play a crucial role in quisinostat-induced anti-NSCLC cells.

Acetylation is essential for p53 activation (Tang et al. 2008). Using western blot analysis, we detected p53 acetylation status in A549 cells by quisinostat treatment. We found that after treatment with quisinostat, the acetylation of p53 at K382/K373 sites was significantly increased (Fig. 6d).

### Quisinostat induced G1 phase arrest and upregulated p21^(Waf1/Cip1)^ expression

**Fig. 7:**
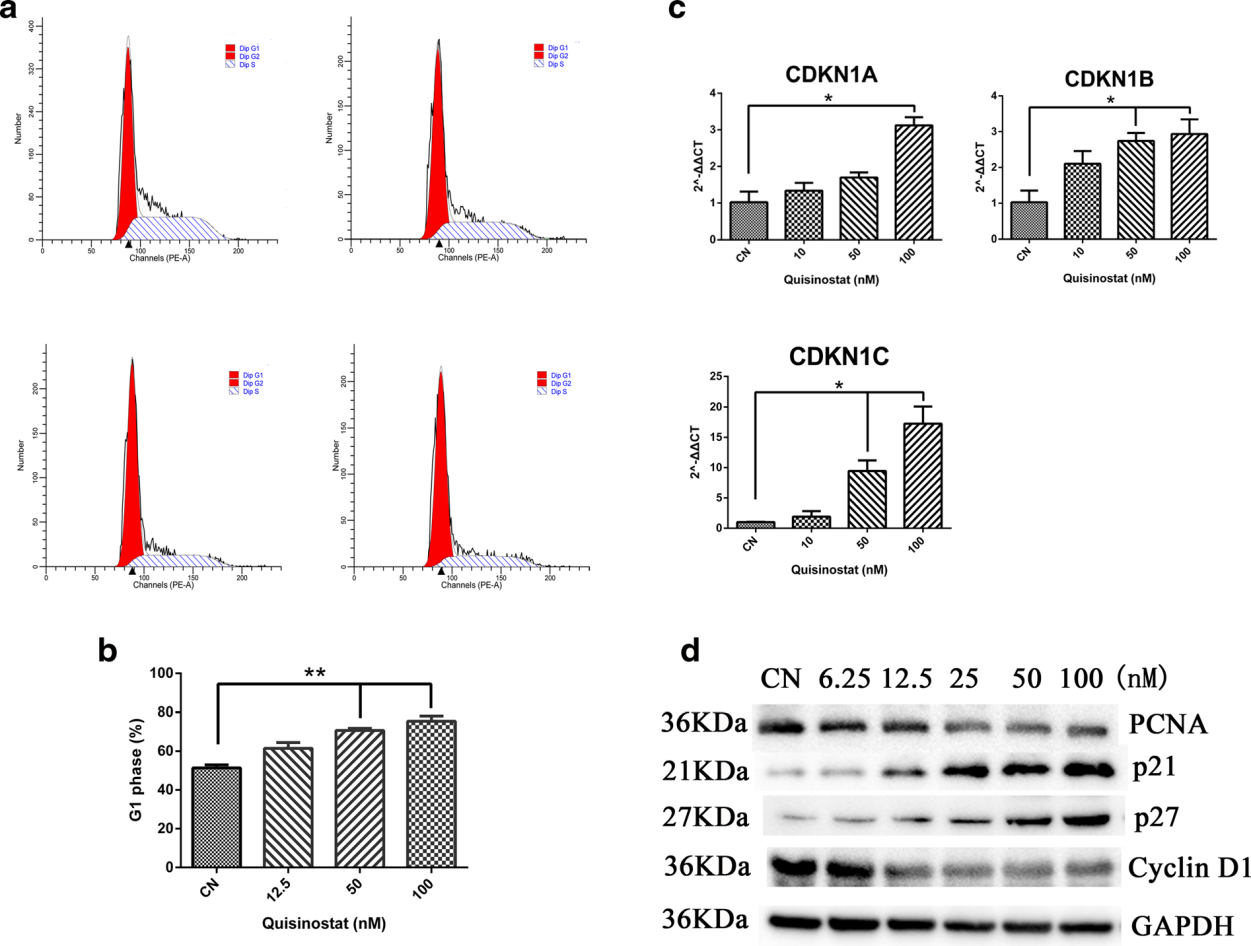
Cell cycle distribution of A549 cells in vitro. **a**, **b** Quisinostat induced cell cycle arrest at G1 phase in dose-dependent manners. Cells were treated with 12.5, 50, and 100 nm of quisinostat for 24 h, respectively, and then, the cells were harvested to determine cell cycle distribution by flow cytometry. **c** CDKN1A, CDKN1B, and CDKN1C were assessed by real-time PCR. **d** Protein expression of PCNA, p21, p27, Cyclin D1, and GAPDH was assessed by western blot. Data are shown as mean ± SD, *n* = 3. *p < 0.05, **p < 0.01 versus control group

The gene expression profiling analysis indicated that quisinostat might affect cell cycle of A549 cells. Our results showed that the cell cycle distribution was markedly changed with quisinostat treatment (Fig. 7a); the amount of cells in G1 phase showed a significant increase (Fig. 7b). To elucidate the mechanism behind the induction of G1 phase arrest, we assessed the effect of quisinostat on the induction of p21^(Waf1/Cip1)^, p27, and p57, the cyclin-dependent kinase (CDK) inhibitors, which are known to regulate the entry of cells at the G1-S phase transition checkpoint (Arts et al. 2009). Real-time PCR revealed that CDKN1A, CDKN1B, and CDKN1C genes, which encode p21^(Waf1/Cip1)^, p27, and p57, respectively, were significantly increased (Fig. 7c). Western blot analysis revealed that quisinostat treatment of the cells resulted in a marked induction of p21^(Waf1/Cip1)^ and p27 in a dose-dependent manner (Fig. 7d). We also found that quisinostat treatment of the cells resulted in a dose-dependent decrease in protein expressions of cyclin D1 and proliferation cell nuclear antigen (PCNA) (Fig. 7d). PCNA is the cell proliferation marker.

### Effects of quisinostat on other lung cell lines

**Fig. 8:**
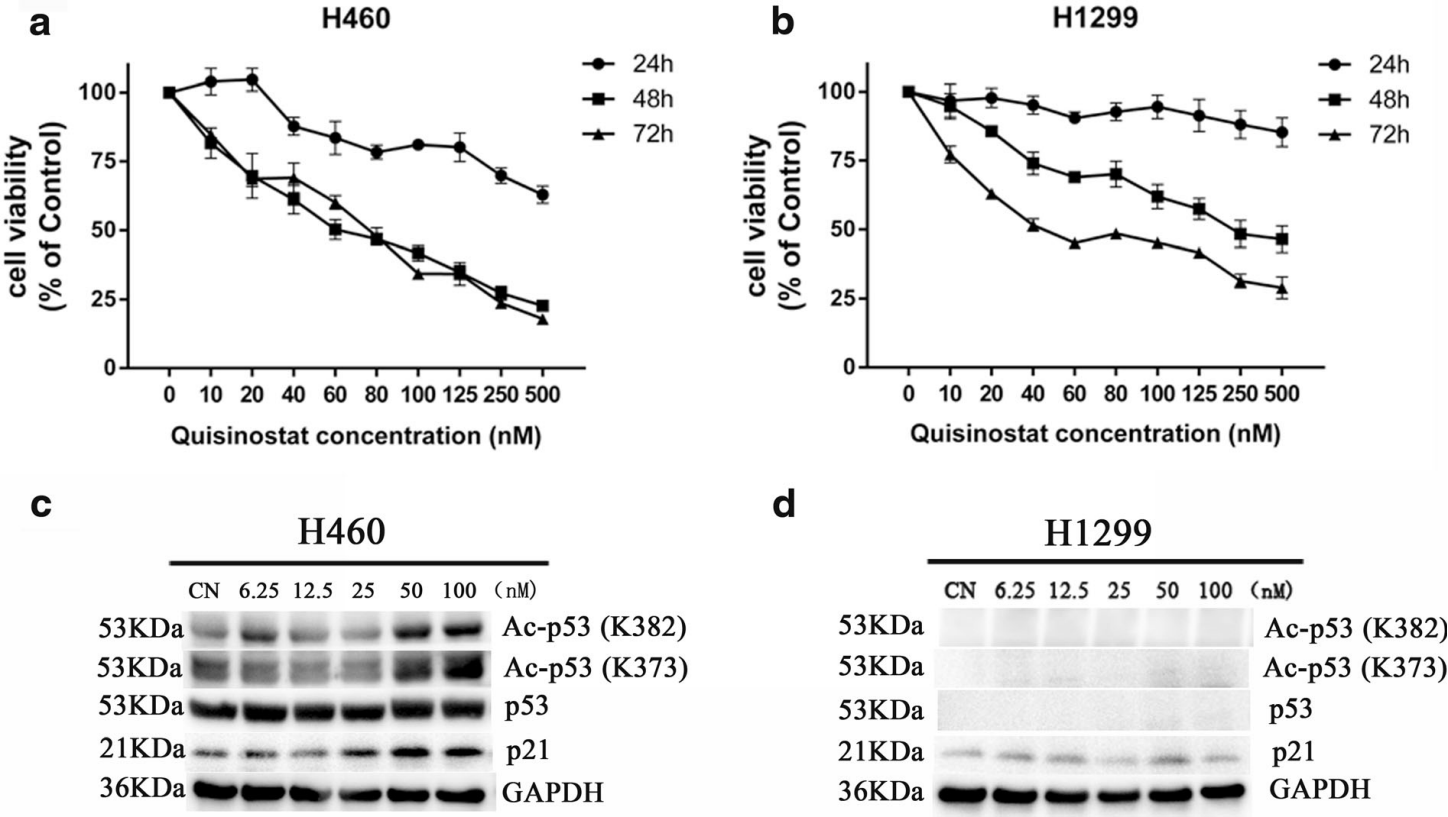
Effects of quisinostat on H460 and H1299 cells. H460 cells (**a**) and H1299 cells (**b**) were treated with series of quisinostat for 24, 48, or 72 h, and cell viability was evaluated. Mean ± SD of three experiments performed in triplicate is shown. **c**, **d** H460 cells and H1299 cells were treated with quisinostat for 24 h. Protein expression of acetylated p53 (Ac-p53), p53, p21, and GAPDH was assessed by western blot

As our study demonstrated that quisinostat treatment of A549 cells resulted in p53 up-acetylation, we further examined the effects of quisinostat on H460—a wild-type p53-expressed NSCLC cell line—and H1299—a p53-defective NSCLC cell line. Cell viability assay showed that quisinostat significantly inhibited cell proliferation of H460 cells (Fig. 8a) and H1299 cells (Fig. 8b) in dose- and time-dependent manners. Compared with A549 (Fig. 1b) and H460, we found that the anti-tumor activity of quisinostat on H1299 is less effective, especially at 24- and 48-h time points. It indicated that A549 and H460 cells are more sensitivity to quisinostat than H1299 cells, which is p53-defective. Using western blot analysis, we assessed the effect of quisinostat treatment on the protein expression of p53 acetylation in H460 cells, and we found that quisinostat up-acetylated p53 protein at K382/K373 as well as A549 cells and significantly increased the expression of p21^(Waf1/Cip1)^ (Fig. 8c). While in H1299 cells, the expression level of p21^(Waf1/Cip1)^ has not been altered by quisinostat treatment (Fig. 8d). These results strongly suggest that quisinostat affects p53 activation in NSCLC cells. In addition, quisinostat also inhibited cell proliferation of another NSCLC cell line SPCA-1 (Supplement Fig. 2a) and human bronchial epithelial cell line HBE (Supplement Fig. 2b).

## Discussion

HDACis have shown therapeutic efficacy in solid and hematological tumors as single agents or in combinations (Kelly and Marks 2005). So far, vorinostat (SAHA) in 2006 and romidepsin (depsipeptide, FK288) in 2009 have been approved by the US Food and Drug Administration (FDA) for the treatment of patients with cutaneous T cell lymphoma (CTCL) (Grant et al. 2010; Mann et al. 2007). It is reported that the profiles of acetylome and proteome were changed by HDACi treatment in NSCLC cells (Wu et al. 2015; Wu et al. 2013), suggesting that NSCLC cells are sensitive with HDACi. Furthermore, clinical trials have demonstrated that HDACis strongly enhance therapy efficacy of EGFR inhibitors (Gerber et al. 2015) and platinum-based chemotherapy (Ramalingam et al. 2010) in NSCLC patients. However, the applicability of HDACi against patients with NSCLC is still limited.

Quisinostat has been documented to exhibit improved pharmacodynamic properties over HDAC inhibitory compounds (Arts et al. 2009). In vitro, quisinostat has demonstrated a potent anti-proliferative activity against multiple human tumor cell lines (Arts et al. 2009). In the present study, we showed that quisinostat significantly inhibited A549 cell proliferation in a nanomolar concentration in dose- and time-dependent manners (Fig. 1b). Previous researchers found that quisinostat induced sustained histone acetylation in normal tissues as well as tumors (Arts et al. 2009). Here, we used a pan-anti-acetyllysine monoclonal antibody to detect acetylated proteins, and we found that quisinostat extremely changed protein acetylation patterns of A549 cells (Fig. 2a). After quisinostat treatment, acetylated histones H3 and H4 were significantly increased in a dose-dependent manner (Fig. 2b), suggesting that quisinostat might affect class I HDACs (HDAC1, HDAC2, HDAC3, and HDAC8). In addition to histones, acetylated α-tubulin (K40) was also markedly increased (Fig. 2b), which is a substrate of HDAC6 (Hubbert et al. 2002), indicating that quisinostat could influence HDAC6 activity. HDAC6 inhibition has been proposed as the molecular basis for synergism of HDACi with other anti-cancer agents that act through proteasome/aggresome deregulation (Bali et al. 2005; George et al. 2005). So, HDAC6 inhibition in A549 cells might improve quisinostat anti-tumor activity. Thus, we can confirm that quisinostat affected HDAC activities and extremely changed protein acetylation patterns of A549 cells.

Apoptosis is a major mechanism to eliminate cancer cells (Green and Kroemer 2004). Our results showed that quisinostat induced apoptosis in A549 cells (Fig. 5a, b). In the signaling transduction of apoptosis, cellular mitochondria play pivotal roles (Jin and El-Deiry 2005). An increase of ROS production and, consequently, loss of ΔΨm were reported as typical phenomena in the process of mitochondria-mediated early apoptosis (Vaux and Korsmeyer 1999). Loss of ΔΨm induces the release of pro-apoptotic factors, such as Cyto c, which is released from the mitochondrial inner space to cytosol (van Loo et al. 2002). Releasing Cyto c can activate caspase-9, which, in turn, activates executioner caspase-3 via cleavage induction (Green 2005). Confocal and flow cytometry data indicated that quisinostat significantly increased cellular ROS generation and destroyed ΔΨm of A549 cells (Fig. 4). Using western blot analysis, we found that the protein expression of Cyto c was increased and caspase cascade was activated by quisinostat (Fig. 5c), suggesting that quisinostat activates mitochondria-mediated intrinsic apoptotic pathway. Recently, cellular ROS was also shown to induce apoptosis by regulating the Bcl-2 family proteins (Li et al. 2004), resulting in increased expressions of pro-apoptotic proteins and decreased expressions of anti-apoptotic proteins. Indeed, the expressions of pro-apoptotic proteins Bax and Bim were significantly increased, while those of anti-apoptotic proteins Bcl-2 and Bcl-xl were decreased by quisinostat treatment (Fig. 5c). These results indicated that quisinostat induced A549 cell apoptosis possibly via a mitochondrial pathway.

EMT is essential for development and physiological response to injury (Thiery et al. 2009). However, under pathological conditions, EMT causes uncontrolled tissue repair and organ fibrosis, as well as the induction of tumor growth, angiogenesis, and metastasis in the context of cancer progression. Epigenetic mechanisms play important roles in EMT-associated processes (Kiesslich et al. 2013), and several classical HDACi completely suppressed EMT process and cell migration (Chen et al. 2013; Kaimori et al. 2010). We found that quisinostat could potently upregulate the expression of E-cadherin (Fig. 3e), which is suppressed by HDAC1 (Ohira et al. 2003); the result is consistent with previous studies (Arts et al. 2009). In NSCLC, loss of E-cadherin expression is a critical event associated with cancer metastasis as well as drug resistance to apoptosis (Kakihana et al. 2009; Onder et al. 2008). Quisinostat restoring E-cadherin expression by inhibiting HDAC activity might increase the sensitivity of NSCLC cells to EGFR inhibitors (Witta et al. 2006), providing a molecular basis for combination therapy in patients with NSCLC. We also found that quisinostat was capable of reducing the expression of N-cadherin and Vimentin (Fig. 3e), leading to a significant inhibition in cell migratory potentials (Fig. 3). Together, our results showed that quisinostat could significantly inhibit EMT process and suppress A549 cell migration.

Our results indicated that quisinostat induced cell apoptosis and inhibited EMT process in A549 cells, but these pathways are non-specific. To search for specific pathway of quisinostat on NSCLC cells, we performed gene expression profiling. Bioinformatics analysis revealed that quisinostat might affect cell cycle progression (Fig. 6a) and activate p53 signaling pathway (Fig. 6c) in A549 cells. A549 expresses wild-type p53 gene, so two possible mechanisms for quisinostat via p53 signaling pathway are increased expression of p53 and activation of p53 functions. In this study, p53 protein level was changed little by quisinostat treatment, whereas the expression of CDKN1A gene, one downstream target gene of p53, was significantly increased (Fig. 7c), as well as at protein level (Fig. 7d), indicating that p53 was activated in A549 cells. Acetylation modification is essential for p53 activity (Tang et al. 2008), implicating in transcriptional activation (Sakaguchi et al. 1998), senescence (Pearson et al. 2000), and chromosome fragility (Yu et al. 2000). Up-acetylated p53 is suggested to play important roles in HDACi treatment on NSCLC cells (Luo et al. 2000). Western blot data showed that quisinostat significantly up-acetylated p53 at K382/K373 sites in A549 cells (Fig. 6d). Up-acetylation of p53 improved the expression of p21^(Waf1/Cip1)^ (Fig. 7d) (Szak et al. 2001), which negatively regulates cell cycle progression combined with other members of CDK inhibitors, including p27 and p57 (Fig. 7c, d) (Donjerkovic and Scott 2000). Previous study has demonstrated that p53 acetylation is responsible for p21^(Waf1/Cip1)^ expression (Tang et al. 2006); substitution of lysine residues 370, 372, 373, 381, and 382 of wild-type p53 with arginine resulted in an 80 % reduction of p21^(Waf1/Cip1)^ inducibility with HDACi treatment (Luo et al. 2000). Upregulation of p21^(Waf1/Cip1)^ has stimulative inhibitory effect on cyclin D1 expression (Fig. 7d), inhibiting the G1-to-S phase transition (as assessed by DNA cell cycle analysis, Fig. 7a). Our results suggest that quisinostat activates p53 by up-acetylating NSCLC cells and therefore improves the expression of p21^(Waf1/Cip1)^, inducing G1 cell cycle arrest.

To make our conclusions more reasonable, we further tested the effects of quisinostat on H460 and H1299 cells. H460 is one kind of NSCLC cells with wild-type p53 gene, while H1299 is a p53-defective NSCLC cell line. When exposed to quisinostat, the viabilities of H460 and H1299 cells were both inhibited (Fig. 8a, b). However, the inhibitory rate of H1299 (Fig. 8b) was lower than that of H460 (Fig. 8a) and A549 (Fig. 1b) at the same concentration, suggesting that quisinostat on H1299 without p53 gene was less effective. Western blot data showed that quisinostat up-acetylated p53 at K382/K373 sites in H460 cells (Fig. 8c) as well as A549 cells and significantly increased the expression of p21^(Waf1/Cip1)^ in a dose-dependent manner (Fig. 8c). In H1299 cells, the expression level of p21^(Waf1/Cip1)^ has not been significantly altered by quisinostat treatment cause of p53 defective (Fig. 8d). Moreover, Luo et al. found that the apoptosis of H1299 increased when the cell was transfected with wild-type p53 and treated with trichostatin A (TSA), a classical HDACi, which inhibits deacetylation of 373 and/or 382 lysine residues of p53, causing an increase of p21^(Waf1/Cip1)^ expression (Luo et al. 2000). That explains a part of reasons why A549 and H460 are more sensitive to quisinostat. Taken together, these results indicate that p53 acetylation is crucial for quisinostat-mediated anti-tumor activity in lung cancer.

In summary, quisinostat exerted strong anti-tumor effects on NSCLC cells related to induction of apoptosis via mitochondrial apoptotic pathways. With inhibition of EMT process, quisinostat significantly reduced cell migration. Up-acetylation of p53 at 382/373 lysine residues has a significant, dose-dependent effect on improving p21^(Waf1/Cip1)^, inducing G1 phase arrest. The detailed unraveling of quisinostat-mediated effects will be helpful for a targeted use in NSCLC and for further selection of suitable combination partners.

## Electronic supplementary material

Below is the link to the electronic supplementary material.

Supplement Figure 1 Differential expressed genes in p53 pathway. A549 cells were treated with quisinostat for 24 h, and gene expression profiling was performed. This picture of p53 signaling pathway is downloaded from KEGG database, the red genes in pathway represent differential expressed genes, while the green genes represent unchanged genes. (GIF 384 kb)

High resolution image (TIF 67 kb)

Supplement Figure 2 Effects of quisinostat on the viability of SPCA-1 and HBE cells. (GIF 152 kb)

High resolution image (TIF 312 kb)
