# Supplementary figures and images for: Histone deacetylase inhibitor induces cell apoptosis and cycle arrest in lung cancer cells via mitochondrial injury and p53 up-acetylation
Source: Cell Biol Toxicol. 2016 Jul 16;32(6):469–82. doi: 10.1007/s10565-016-9347-8 (PMC5099365; doi:10.1007/s10565-016-9347-8)

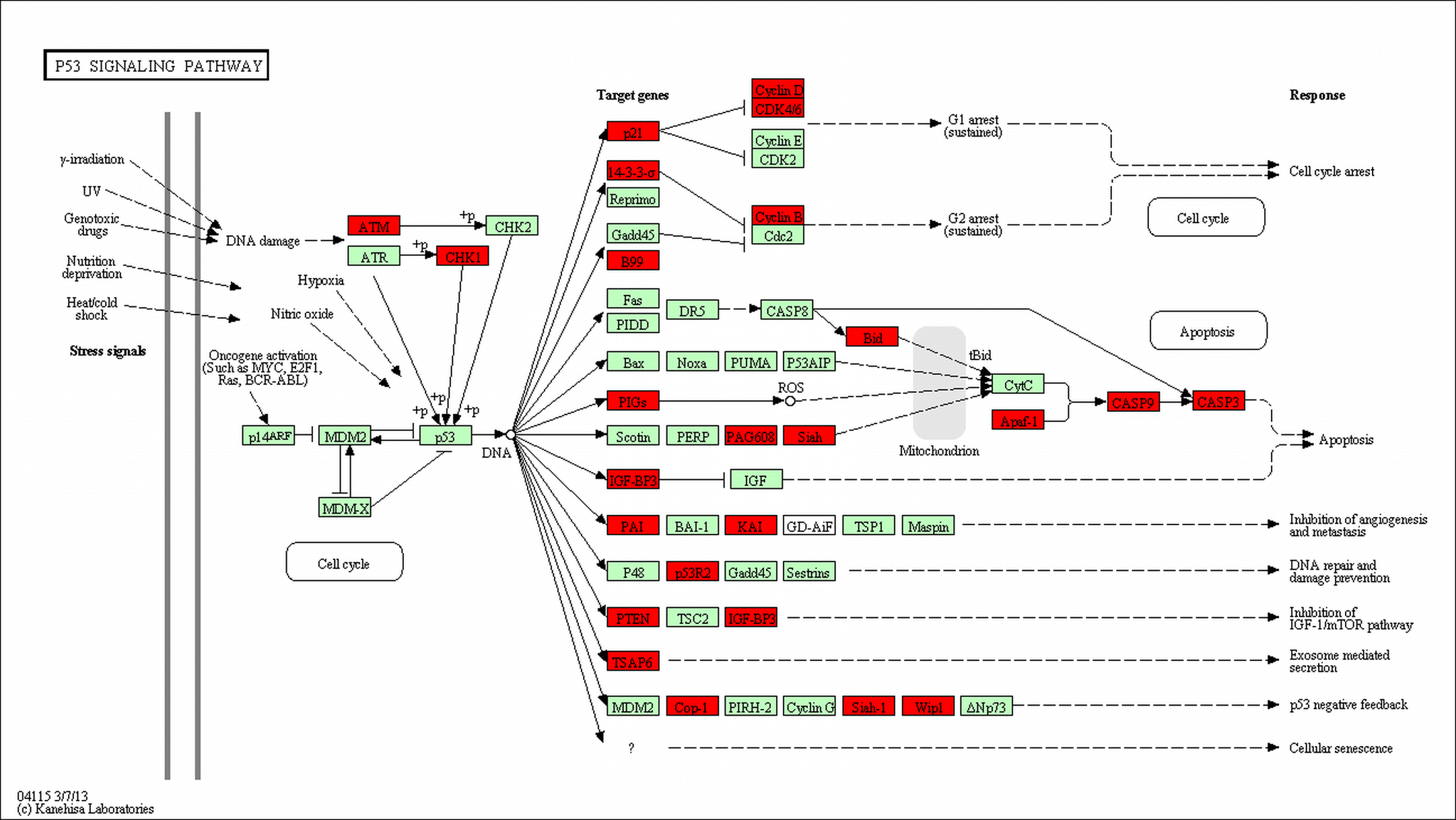

Supplement: Supplementary file 1 — Differential expressed genes in p53 pathway. A549 cells were treated with quisinostat for 24 h, and gene expression profiling was performed. This picture of p53 signaling pathway is downloaded from KEGG database, the red genes in pathway represent differential expressed genes, while the green genes represent unchanged genes. (GIF 384 kb) [file 10565_2016_9347_Fig9_ESM.gif]

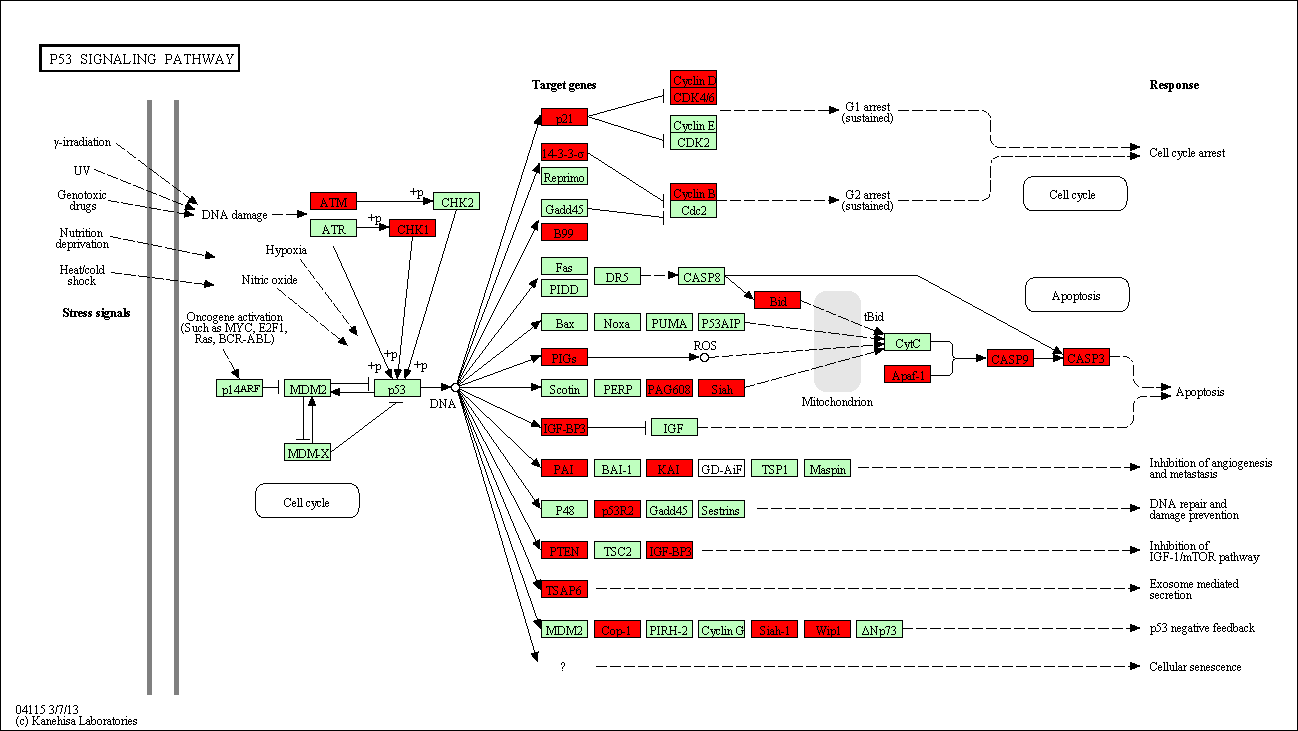

Supplement: Supplementary file 2 — High resolution image (TIF 67 kb) [file 10565_2016_9347_MOESM1_ESM.tif]

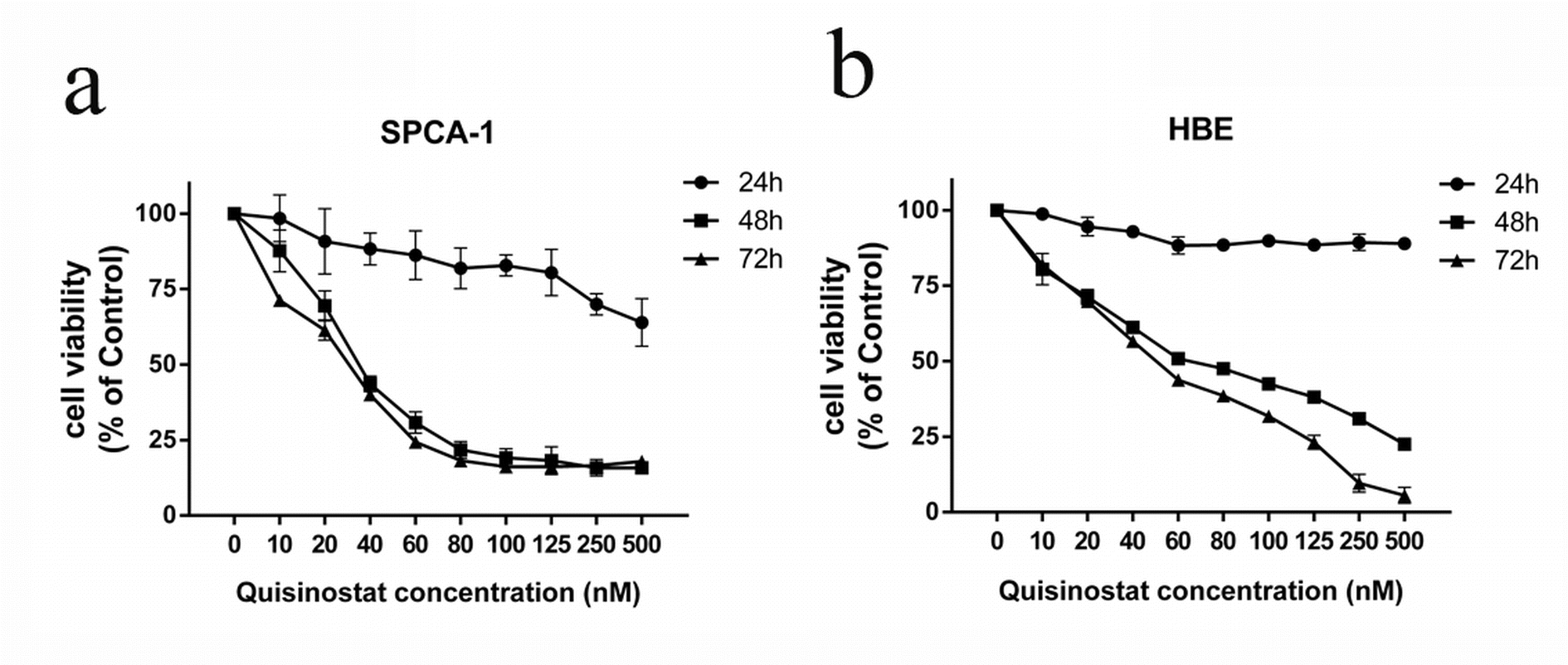

Supplement: Supplementary file 3 — Effects of quisinostat on the viability of SPCA-1 and HBE cells. (GIF 152 kb) [file 10565_2016_9347_Fig10_ESM.gif]

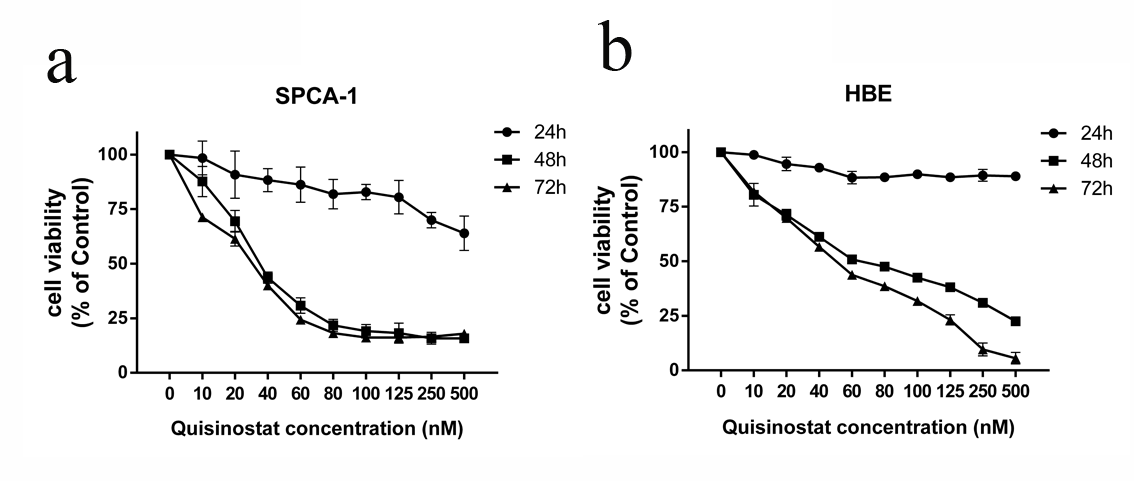

Supplement: Supplementary file 4 — High resolution image (TIF 312 kb) [file 10565_2016_9347_MOESM2_ESM.tif]
